# Minimum Intracanal Dressing Time of Triple Antibiotic Paste to Eliminate Enterococcus Faecalis (ATCC 29212) and Determination of Minimum Inhibitory Concentration and Minimum Bactericidal Concentration: An Ex Vivo Study

**Published:** 2018-01

**Authors:** Sholeh Ghabraei, Mohammad Marvi, Behnam Bolhari, Parisa Bagheri

**Affiliations:** 1 Assistant Professor, Dental Research Center, Dentistry Research Institute, Tehran University of Medical Sciences, Tehran, Iran; Department of Endodontics, School of Dentistry, Tehran University of Medical Sciences, Tehran, Iran; 2 Dentist, Private Practice, Tehran, Iran; 3 Associate Professor, Laser Research Center of Dentistry, Dentistry Research Institute, Tehran University of Medical Sciences, Tehran, Iran; Department of Endodontics, School of Dentistry, Tehran University of Medical Sciences, Tehran, Iran; 4 Postgraduate Student, Department of Endodontics, School of Dentistry, Tehran University of Medical Sciences, Tehran, Iran

**Keywords:** Calcium Hydroxide, Doxycycline, Enterococcus Faecalis, Metronidazole

## Abstract

**Objectives:**

Enterococcus faecalis (E. faecalis) is the most commonly isolated microorganism from teeth with postoperative infection. Triple antibiotic paste (TAP) has the ability to eradicate microorganisms from the root canal system when used as an intracanal medicament. The aim of this study was to determine the minimum duration of application of TAP required for elimination of E. faecalis from the root canal system and its minimum inhibitory concentration (MIC) and minimum bactericidal concentration (MBC) in an ex-vivo model.

**Materials and Methods:**

Root canals of 34 extracted human single canal teeth were inoculated with E. Faecalis after instrumentation, and then 4 g of TAP (ciprofloxacin, metronidazole and doxycycline) was mixed with 4.5 mL of saline and applied as intracanal medicament. The teeth were sectioned longitudinally and dentin chips were collected and evaluated to determine the count of bacterial colonies. Micro-dilution broth test was used to assess the MIC and MBC of TAP. Data were analyzed using SPSS version 22 via the Wilcoxon signed rank test.

**Results:**

After seven days of application of TAP as intracanal medicament, E. faecalis was eliminated from the dentinal tubules of the apical half of root canal up to 400 μ depth. The MIC and MBC of TAP in its original concentration were both found to be 16 μg/mL.

**Conclusions:**

The original concentration of TAP was found to be 5×10^4^ times its MIC. Considering the risk of coronal discoloration of teeth following the use of TAP, application of its lower concentrations is recommended.

## INTRODUCTION

Presence of microbes in the root canal system is the main cause of apical periodontitis with endodontic origin [1]. Mechanical cleaning along with the use of irrigating solutions during endodontic treatment decreases the count of microorganisms in the root canal system. However, in case of secondary infection or treatment-resistant cases that do not well respond to primary endodontic treatment or regenerative treatment of necrotic open apex teeth, application of intracanal medicaments is recommended in-between treatment sessions for complete elimination of microorganisms from the root canal system and to provide a suitable environment for root development and maturation [2–5]. Triple antibiotic paste (TAP) has shown promising antibacterial activity when used as intracanal medicament. Its initial composition was suggested by Hoshino et al, [6] in 1996 and it is produced by mixing minocycline, metronidazole and ciprofloxacin in combination with saline. When used as intracanal medicament in extracted teeth which were clinically diagnosed hopeless due to their extensive caries, it eliminated the microorganisms from the root canal system and dentinal tubules [6,7]. In addition to the suggested application of TAP in the root canal system, it has shown successful clinical results in regenerative treatments and canals with persistent and treatment-resistant infections. Clinicians have used this antibacterial paste for seven to 28 days [2,8–13]. Only few studies have evaluated the proper consistency of this mixture to eliminate microorganisms. Therefore, its accurate consistency is unknown. An in-vitro study reported the optimal concentration of TAP to be 100 μg/mL for sufficient antibacterial activity in the root canal in culture media [6]. Although TAP is successful in elimination of microorganisms when used in a paste-like consistency, coronal discoloration after its application has been commonly reported following regenerative treatments using TAP. This drawback negatively affects the success of these treatments particularly in the esthetic zone despite the presence of other success criteria [11–14]. Therefore, determining the accurate consistency seems to be clinically valuable.

To the best of authors’ knowledge, no previous study has assessed the minimal required duration of application of TAP as intracanal medicament. Thus, one of the goals of this study was to determine the minimal required duration of application of TAP in paste-like consistency as intracanal medicament to eliminate enterococcus faecalis (E. faecalis) from the root canal system in an ex vivo model. In addition, to evaluate the difference between its paste-like consistency and minimum concentration that can inhibit bacterial growth, its minimum inhibitory concentration (MIC) and minimum bactericidal concentration (MBC) were also determined.

## MATERIALS AND METHODS

Thirty-four single canal human teeth which had been extracted for orthodontic or periodontal reasons and stored in saline until the experiment to prevent dehydration, were used in this study. The study was approved in the ethics committee of our university (code:6003). For easier access to the apical third of the root canals, the crowns were cut at the cementoenamel junction using a diamond disc and high speed hand piece. Cleaning and shaping of the root canals were carried out manually using stainless steel hand K files (Mani, Tochigi, Japan). A #15 K-file was introduced into the canal until its tip was visible at the apex. Working length was set at 1 mm shorter than the apex. Apical flaring was performed using #35 file and then the middle and coronal thirds were instrumented using the step-back technique up to #70 file. Root canal irrigation was performed by a syringe containing saline during instrumentation. After cleaning and shaping, the root canal was rinsed with 17% EDTA (Ariadent, Tehran, Iran) for one minute and then with 5.25% sodium hypochlorite (Shemin, Eslamshahr, Tehran, Iran) for one minute for smear layer removal, followed by a final rinse with saline [3]. Teeth with calcified canals, root caries or open apex were excluded and replaced with intact teeth. After root canal preparation, the teeth were placed in 1.5 mL microtubes containing distilled water. The teeth were sterilized with 40 kGy gamma radiation for three hours and 45 minutes.

**Fig. 1:**
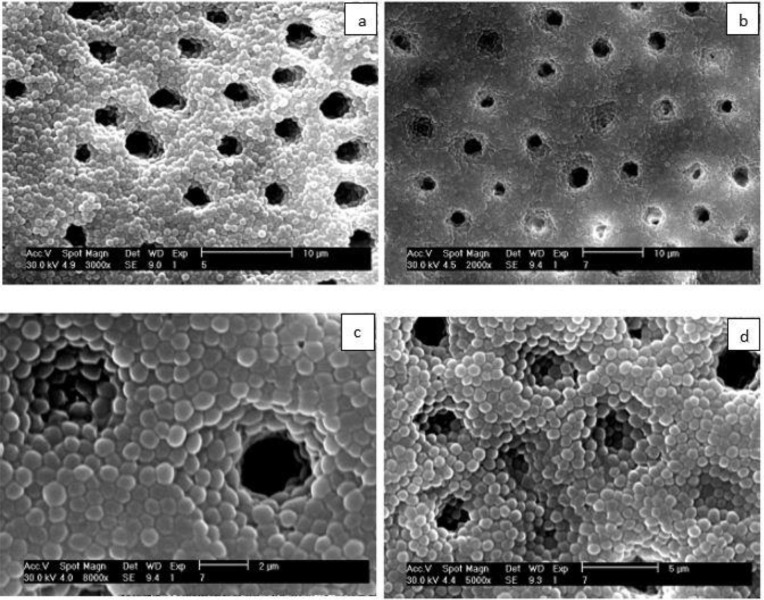
SEM images of E. faecalis biofilm on the root canal walls and inside the dentinal tubules (a) ×2000 maginification, (b) ×3000 magnification, (c) ×5000 magnification, (d) ×8000 magnification

Distilled water was removed from the microtubes containing sterile teeth by using a sterile syringe. Bacterial suspension of E. faecalis (ATCC 29212) with 0.5 McFarland standard concentration containing 1.5×10^8^ colony forming units (CFUs)/mL was prepared in tryptic soy broth (TSB) (Merck, Darmstadt, Germany) and inoculated into the root canals of all teeth except for five teeth that were served as negative controls. The teeth were placed in sterile microtubes containing 1 mL of TSB culture medium and incubated at 37°C for one week. The TBS medium was refreshed every 48 hours. After completion of the incubation period, the teeth were divided into three groups:

Group 1: Twenty-four teeth subjected to TAP/saline (based on the results of a pilot study, CFU counts of this group were evaluated in two series of 12: 12 teeth were evaluated six days and 12 teeth were evaluated seven days after the application of TAP as intracanal medicament).
Group 2: Five teeth serving as positive controls containing only bacteria
Group 3: Five teeth serving as negative controls, which were placed in sterile TSB medium after sterilization without bacterial inoculation. Before placing the TAP inside the root canals, four teeth (other than the samples of the three groups which were not used further in the experiment) that had been inoculated with the bacteria were sectioned horizontally and then in order to confirm the formation of biofilm, the samples were viewed under a scanning electron microscope (SEM; Fig. 1).

**Fig. 2:**
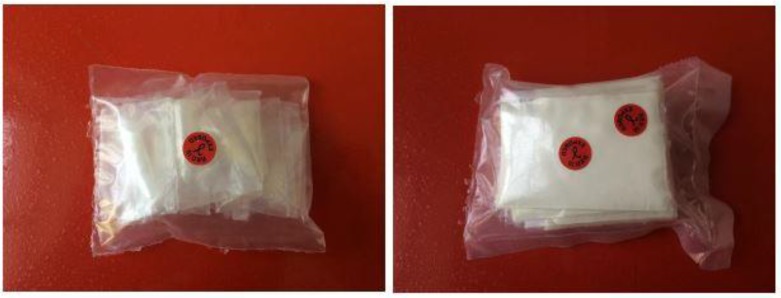
Strilization of teeth and TAP by 90 kGy gamma radiation

The antibiotic powder was prepared by mixing equal portions of metronidazole (Pars Darou, Tehran, Iran), ciprofloxacin (Razak, Tehran, Iran), and doxycycline (Razak, Tehran, Iran) [15]. The mixture was sterilized by 40 kGray gamma radiation (Fig. 2). Intracanal medicament was prepared by mixing 4 g of the powder with 4.5 mL of saline. The coronal and apical openings were sealed with wax. The samples were stored in TBS at 37°C in an incubator, and the TBS was refreshed every 48 hours.

**Fig. 3:**
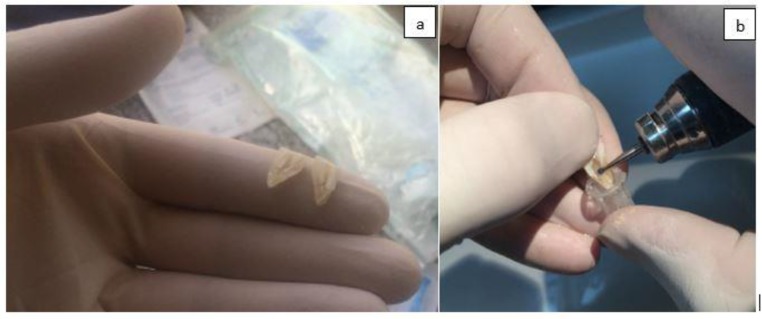
(a) Longitudinal secioning of teeth; (b) scraping of the apical half of root canal walls with a round bur

The samples were longitudinally sectioned after six and seven days and dentin chips were collected by scraping the apical half of the root canal walls with a #4 round bur using the whole diameter of the bur (400 μm depth). In order to prevent cross contamination between samples, each bur was used for only one sample (Fig. 3). The dentin chips were placed in 0.5 mL microtubes. Each 0.5 mL microtube was weighed before and after preparing the dentin chips to calculate the weight of dentin chips obtained from each tooth. A 10-fold dilution was made to count E. faecalis (ATCC 29212) CFUs. Bacterial colony count was evaluated using the method described by Miles et al [16]. The MIC and MBC of TAP were also determined. For this purpose, first 0.5 McFarland standard suspension (containing 1.5×10^6^ CFUs/mL) of E. faecalis (ATCC 29212) was prepared using TSB culture medium and then the following steps were performed:

1. Preparing 1 mg/mL concentration of TAP in cation-adjusted Mueller Hinton broth medium
2. Transferring 50 μL of cation-adjusted Mueller Hinton broth medium to wells #2 to #11 of a microplate under sterile conditions (well #11 served as the negative control)
3. Adding 100 μL of the TAP solution with 1 mg/mL concentration to well #1.
4. Transferring 50 μL of TAP solution from well #1 to well #2, to dilute it by half. Serial dilution was continued to the 10^th^ well and finally 50 μL of the contents of the well #10 was extracted. By doing so, wells #1 to #10 had different concentrations of serially diluted TAP (each well had half of the concentration of TAP in its previous well).
5. The 2^nd^ to the 10^th^ wells had ½ to 1/1024 concentration of TAP, respectively.
6. Next, 50 μL of the E. faecalis (ATCC 29212) suspension was added to wells #1 to #10 (well #11 was the negative control and received no bacterial inoculation).
7. To determine the MBC of TAP, 50 μL of the solution was removed from the well before the MIC well, and the well after the MIC well. The solution was inoculated into tryptic soy agar plate and incubated at 37°C for 24 hours.

### Statistical analysis:

Data were collected and analyzed using SPSS version 22 (SPSS Inc., IL, USA) via the Wilcoxon signed rank test.

The level of statistical significance was set at P<0.05.

## RESULTS

Tables 1 and 2 show the E. faecalis (ATCC 29212) colony count in the TAP group and the positive control group.

**Table 1.** Effect of TAP on bacterial colony count after six and seven days

**Tooth number**	**Six days**	**Seven days**
1	1.2 * 10^3^	0
2	1 * 10^3^	0
3	2.3 * 10^3^	0
4	2.8 * 10^3^	0
5	1.7 * 10^3^	0
6	2.9 * 10^3^	0
7	1.1 * 10^3^	0
8	1 * 10^3^	0
9	2.4 * 10^3^	0
10	1.8 * 10^3^	0
11	2.4 * 10^3^	0
12	1.7 * 10^3^	0

**Table 2.** E. faecalis (ATCC 29212) colony count in the positive control group

**Tooth number**	**Weight of dentin chips**	**Weight scale**	**E. Faecalis colony count**
1	0.009	0.008	4.72 × 10^4^
2	0.008	0.008	6.2 × 10^4^
3	0.019	0.008	5.64 × 10^4^
4	0.029	0.008	3.8 × 10^4^
5	0.016	0.008	4.26× 10^4^

Mean colony count in the positive control group at the end of study = 4.924× 10^4^

On day six, TAP showed significant antibacterial activity and E. Faecalis (ATCC 29212) count decreased. On day seven, E. Faecalis (ATCC 29212) was completely eliminated in all dentin chips obtained from the apical half of the root canals up to 400 μm depth (P<0.001, Table 1).

The results showed that bacteria remained viable in the biofilm formed on the root canal wall despite the coronal and apical seal in the positive control group without intracanal medicament (P<0.001, Table 2).

In the five negative control teeth, dentin chips obtained from the apical half of the root canals showed absence of E. faecalis (ATCC 29212) in tryptic soy agar (P<0.001).

The MIC of TAP was measured to be 16 μg/mL. No growth in the MIC well and the well before that and bacterial growth in the well after the MIC well indicated equality of MBC and MIC of TAP.

## DISCUSSION

E. faecalis is the most commonly isolated microorganism from teeth with failed endodontic treatment. It is often resistant to antibacterial agents [3, 17, 18]. Thus, it was used as the target microorganism in our study. Also, since the bacteria in biofilm are more resistant than free bacteria [3,17], E. faecalis in its biofilm was evaluated in our study. The fact that E. faecalis could be cultured in the positive control group confirmed that the heat generation of high speed hand piece did not cause bacterial death.

Since minocycline was not available, doxycycline was used in the composition of TAP in our study. TAP was prepared using equal amounts of ciprofloxacin, metronidazole and doxycycline. Doxycycline has bacteriostatic efficacy similar to that of minocycline and it has been shown that bacteria present in apical periodontitis lesions are sensitive to both of these antibiotics [19]. Although the American Association of Endodontists has recommended the use of TAP in the range of 0.1–1 mg/mL for 1 to 4 weeks [8], there is no document regarding the exact concentration and duration of TAP for use as an intracanal medicament. Previous case reports have used TAP as intracanal medicament for seven to 28 days [2, 9–13, 20, 21]. In contrast to other studies [22–26], we used this antimicrobial mixture as an intracanal medicament.

In our study, the standard method by the Clinical and Laboratory Standard Institute was used for the calculation of MIC and MBC of TAP. Adl and Motamedifar [19] in 2012 used another method for determination of MIC and MBC. Thus, the results of the two studies could not be compared. Sabrah et al, [15] in 2013 calculated the MIC and MBC of TAP (metronidazole, ciprofloxacin and minocycline) and double antibiotic paste (DAP; metronidazole and ciprofloxacin) using 2-fold dilution method and reported the MIC and MBIC of TAP for E. faecalis biofilm to be 0.003 mg/mL. The MBC of TAP for E. faecalis was reported to be 0.3 mg/mL. The MIC and MBIC of DAP were found to be 0.001 mg/mL for E. faecalis and the MBC of DAP was reported to be 0.14 mg/mL. The formation rate of bacterial biofilm decreased in presence of all concentrations of TAP and DAP and this reduction was not significantly different between TAP and DAP.

It should be noted that the amounts of MIC and MBC that were calculated in this study could have been different if other microorganisms had been inoculated into the root canals, but we used E. faecalis biofilm due to the reasons mentioned earlier.

Since one of the objectives of this study was to find the minimum required duration of application of TAP for elimination of E. faecalis, in a pilot study we collected samples from 400 μ depth of root canal wall at 24 hours, three days, five days and seven days. Since the bacterial culture became positive at five days and negative at seven days after the application of TAP, it was decided to assess the six-day and seven-day time points in the main study, and 12 teeth were allocated for assessment of bacterial count at each time point. At six days, TAP showed significant antibacterial activity and it completely eliminated E. faecalis biofilm at seven days.

The apical half of the root canals was selected for collection of dentin chips considering the significance of cleaning of the apical region during endodontic treatment, difficult access to this region and the fact that the amount of bacterial biofilm in the apical part of root canal is higher in both endodontically treated and untreated teeth [22].

Sato et al. [7] evaluated the antibacterial efficacy of TAP against Escherichia coli (E. coli) in the root canal system and concluded that it successfully eliminated the target bacteria from the root canal system after 48 hours. Standard strain E. faecalis (29212) was used in our study, which was different from the E. coli evaluated by Sato et al [7]. This difference explains the controversy in the results of the two studies. Also, the current study assessed the effect of TAP on bacterial biofilm. Moreover, in our study, dentin chips were collected by removing 400 μ of the root canal wall by a #4 round bur while Sato et al. [7] used Gates Glidden drills to superficially remove dentin from the root canal walls.

Saber and El-Hady [23] in their in vitro study showed that application of co-amoxiclav, ciprofloxacin, clindamycin and doxycycline as intracanal medicament in the root canals of 148 single canal teeth for one week resulted in elimination of E. faecalis biofilm from the root canal system. They found no significant difference in the efficacy of the tested antibiotics for elimination of bacterial biofilm. In our study, TAP remained in the canal for seven days, as in the study by Saber and El-Hady [23] and successfully eliminated E. faecalis biofilm from the root canal system. Moreover, in our study, dentin chips were obtained by scraping the root canal walls with 400 μ depth using a #4 round bur while Saber and El-Hady [23] collected dentin chips by scraping the root canal walls using #2 Gates Glidden drill. In the current study, TAP was prepared in paste-like consistency and applied into the canals. The exact amount of powder and liquid used was also recorded for the purpose of reproducibility of paste preparation with the same concentration. The concentration of TAP used in our study was 5×10^4^ times its MIC concentration against E. faecalis. Use of high concentrations of TAP can cause complications such as coronal discoloration of teeth.

Regarding the discoloration caused by TAP, which is composed of ciprofloxacin, metronidazole and minocycline, Kim et al. [14] showed that minocycline was the main cause of tooth discoloration due to TAP. Thus, it has been suggested to use other antibiotics such as cefaclor [23, 24], fosfomycin [24], amoxicillin [13, 20, 24], or Augmentin [12] instead of minocycline or DAP (ciprofloxacin and metronidazole) [25] instead of the classic TAP [21]. In another study, Kahler et al. [13] indicated that despite replacement of minocycline with amoxicillin, 10 out of 16 teeth that underwent regenerative treatment showed coronal discoloration. Also, replacement of minocycline with cefaclor and doxycycline in another study did not prevent visible coronal discoloration of teeth following application of these drugs for three weeks [25]. Thus, strategies to prevent coronal discoloration following the use of antibiotic compounds must be studied in further details. However, TAP is one of the most commonly used intracanal medicaments for disinfection in regenerative endodontic procedures and is one of the suggested medications in the American Association of Endodontists guidelines. Considering the risk of coronal discoloration following the use of TAP, future studies are required to assess the antibacterial effects of lower concentrations of TAP on the biofilm. Lower concentrations of TAP have easier clinical application and better penetration into the root canal system, although they can be washed from the root canal system and need a scaffold. Also, production of nano-TAP can be an interesting topic for future research. It should be noted that considering the critical role of stem cells in endodontic regenerative treatments and the coronal discoloration potential of TAP, future studies are required to find a medicament with the least side effects for this treatment protocol.

## CONCLUSION

Based on the results of this study, the concentration of TAP which is clinically used, was found to be 5×10^4^ times its MIC. Considering the risk of coronal discoloration of teeth following the use of TAP, application of its lower concentrations is recommended. Our results suggest that the minimum duration of application of TAP required for elimination of E. faecalis from the root canal system is seven days; however; it should be noted that this was an in vitro study using one specific microorganism; therefore, further clinical studies are recommended.
